# Intrauterine fetal death followed by shoulder dystocia and birth by modified posterior axillary sling method: a case report

**DOI:** 10.1186/s12884-021-04126-4

**Published:** 2021-10-03

**Authors:** Angel Hoi Wan Kwan, Annie Shuk Yi Hui, Jacqueline Ho Sze Lee, Tak Yeung Leung

**Affiliations:** Department of Obstetrics and Gynaecology, The Chinese University of Hong Kong, Shatin, Hong Kong SAR

**Keywords:** Shoulder dystocia, Posterior axillary sling, HELPERR Mnemonic, Posterior arm extraction, Case report

## Abstract

**Background:**

Various manoeuvres such as McRoberts position, suprapubic pressure, rotational methods, posterior arm extraction and all-four position (HELPERR) have been proposed for relieving shoulder dystocia with variable success. Posterior axillary sling method using a rubber catheter was proposed in 2009 but has not been widely used. We modified this method using ribbon gauzes and a long right-angle forceps and report a successful case.

**Case presentation:**

A 44 years old parity one Chinese woman with a history of a caesarean delivery and poorly controlled type 2 diabetes mellitus was admitted to the Accident and Emergency Department for advanced stage of labour at term. Upon arrival, intrauterine fetal demise was diagnosed with severe asynclitism causing obstruction at the perineum. Episiotomy resulted in birth of the fetal head. The fetal posterior right shoulder, however, remained very high up in the pelvis and HELPERR methods failed to extract the shoulders. We then tied two long ribbon gauzes together, and guided its knot to the anterior aspect of the posterior axilla. By using a long right-angle forceps (24 cm long) to grasp the knot on the posterior side of the axilla and pulling it through, a sling was formed. Traction was then applied through the sling to simultaneously pull and rotate the posterior shoulder. A stillbirth of 3488 g was finally extracted.

**Conclusions:**

We modified the sling method by using two ribbon gauzes, tied together and a right-angle forceps with several advantages. Compared to a rubber catheter, ribbon gauze with a knot can be easily held between the fingers for easy guidance past the fetal axilla. It is also thin, non-elastic and stiff enough to ensure a good grip for traction. The long and slim design of the right-angle forceps makes it easy to pass through a narrow space and reach the axilla high up in the pelvis. We emphasize simultaneous traction and rotation, so that the shoulders are delivered through the wider oblique pelvic outlet dimension.

**Supplementary Information:**

The online version contains supplementary material available at 10.1186/s12884-021-04126-4.

## Background

Shoulder dystocia is an uncommon and unpredictable obstetric emergency [1]. Improper or delayed management is associated with an increased risk of maternal and fetal morbidity and mortality [2, 3]. Various manoeuvres such as McRoberts manoeuvre, suprapubic pressure, rotational methods, posterior arm extraction and all-fours position have been proposed but their success rates are variable, ranging from 25–50% for McRoberts and suprapubic pressure, to 65–70% for rotational methods and posterior arm extraction [3, 4]. Cluver and Hofmeyr reported in 2009 the use of a rubber catheter in forming a sling underneath the fetal axilla, and successful extraction of the shoulders through traction on the sling [5]. We concur that this posterior axillary sling method is very useful, yet it has not been widely used. We modified this method using ribbon gauzes and a long right-angle forceps, and report here a successful case, and discuss the advantages of our instruments over a rubber catheter.

## Case presentation

A 44 years old Chinese woman, para one with a history of poorly controlled type 2 diabetes mellitus since 2011. In 2013 she underwent induction of labour for poor glucose control at term, and finally gave birth by caesarean for failed induction of labour. The baby was born alive with a birthweight of 3.630 g. Afterwards she defaulted medical follow-up for six years due to financial and childcare issues. She did not have any fertility plan, but did not practice any contraception either.

She was unaware of her index pregnancy, as she had a negative pregnancy test after three months of amenorrhea in mid 2018. She did not seek any medical advice despite remaining amenorrhoic as she thought she was menopausal. She noticed an increase in bodyweight, but never felt any fetal movements. On the day of admission in early 2019, she had leaking sensations and colicky abdominal discomfort at home. As she thought of urinary incontinence and constipation, she started straining in the toilet. Afterwards she felt a bulging mass at her perineum, and noticed that the fetal scalp was already at the introitus, but she could not push the baby out.

She delayed calling an ambulance and arrived three hours later in the Accident and Emergency Department of Prince of Wales Hospital in Hong Kong. Upon arrival, uterine contractions had completely subsided. Maternal vital signs were stable. Physical examination revealed a soft abdomen which was distended with the gravid uterus. The woman was put into the lithotomy position and the edematous fetal scalp was distending the perineum. The fetal head was still within the vagina, facing the maternal left side. Ultrasound examination confirmed a demised term sized fetus. The woman was told to push but in vain as she was exhausted. As the fetus had died for some time with loss of muscle tone, the fetal neck was hyperflexed towards the right side, resulting in severe asynclitism. Hence, after making an episiotomy, the operator had to put his right index and middle fingers into the fetal mouth, and applied force to correct the neck hyperflexion and the head asynclitism. After birth of the head difficult shoulder dystocia occurred. The posterior right shoulder remained very high up in the pelvis. Different manoeuvres including McRobert’s, suprapubic pressure and internal rotational manoeuvres were applied to relieve the shoulders, but all failed. Posterior arm extraction was attempted, but the arm was too high and unreachable by the operator’s hand. The mother was already exhausted and could not get into an all fours position.

**Fig. 1:**
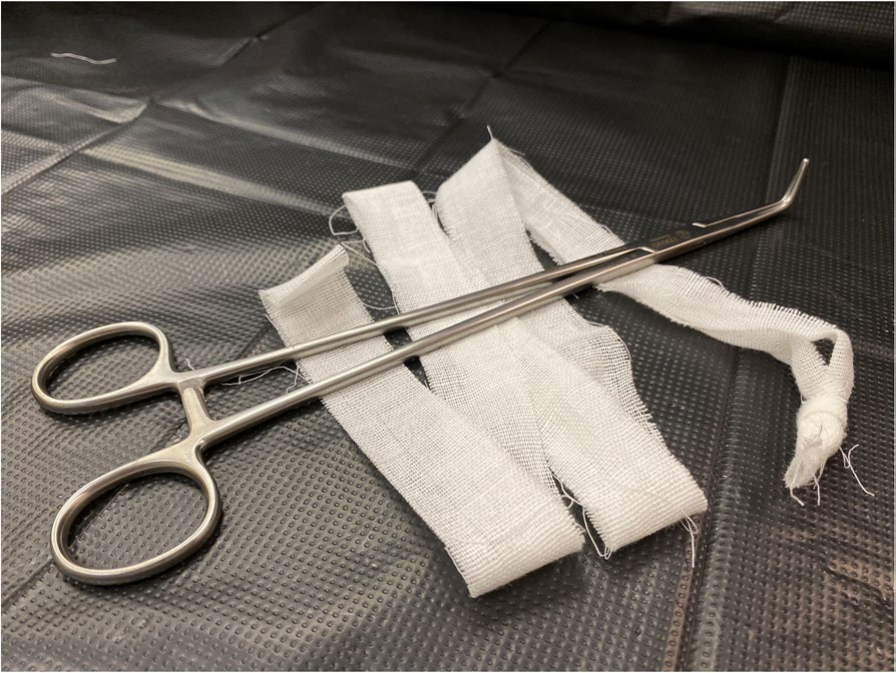
A long right-angle forceps (24 cm in length) and two long ribbon gauzes that are tied together

**Fig. 2:**
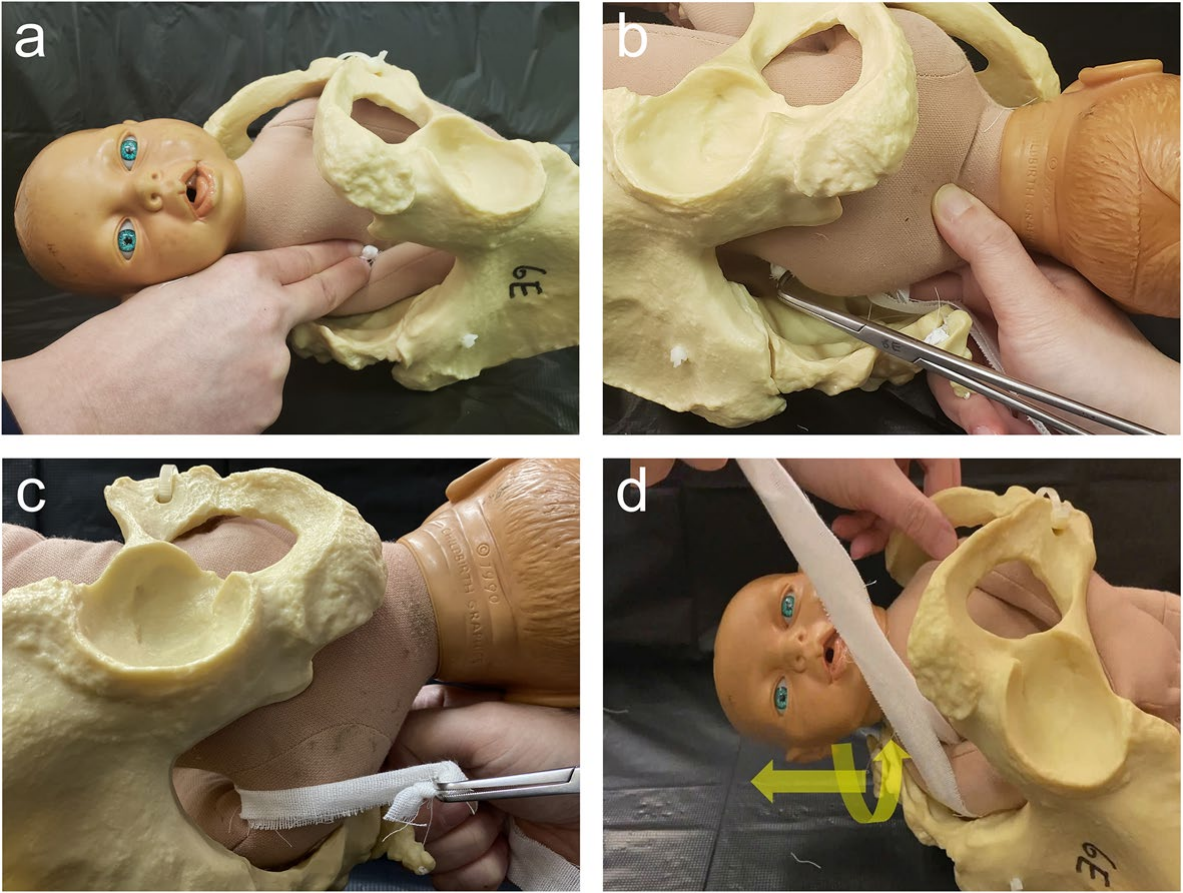
Extraction of the posterior arm with a sling formed by ribbon gauzes and forceps. **a** The knot of the tied ribbon gauzes is guided to the anterior side of the axilla of the posterior shoulder by the operator’s fingers. **b** Forceps is inserted deep into the maternal pelvis, to reach the posterior axilla at the back, and its tips grasp the knot of the ribbon gauzes through the axilla. **c** The ribbon gauze is pulled through the axilla by forceps to create a sling around the axilla. **d** Traction is applied to the ribbon sling in an outward and rotational manner to extract the posterior shoulder

Finally, an attempt to extract the posterior shoulder with posterior axillary sling method was performed (Video 1). Firstly, two long ribbon gauzes were tied together (Fig. 1). With its knot held by the operator’s right index and middle fingers, the ribbon gauzes were brought to the anterior side of the axilla of the posterior right shoulder (Fig. 2a). A pair of long right-angle forceps (Fig. 1) was inserted along the fetal back with the operator’s left hand, so that the forceps tips reached the posterior side of the right axilla. The forceps tips were then passed under the axilla to grasp the knot of the ribbon gauzes (Fig. 2b). The ribbon gauze was then pulled through to create a sling around the axilla (Fig. 2c). Traction force was applied through the ribbon sling, in an outward, leftward (maternal left side) and upward manner in order to pull down the posterior shoulder, as well as to rotate it in anti-clockwise direction (Fig. 2d). The posterior right shoulder was then extracted successfully, followed by the anterior left shoulder and the rest of the baby’s body. There was no additional perineal or vaginal tear, with a total blood loss of 150 ml. The baby weighted 3488 g and was certified stillborn. The woman was managed with uterotonic prophylaxis and antibiotic cover. Placental pathological examination revealed severe acute chorioamnionitis and the placenta swab showed growth of *Escherichia Coli*. Other investigations were all unremarkable.

## Discussion and conclusions

The cause of intrauterine fetal death (IUFD) in this case might be related to suboptimal diabetic control or rupture of membranes with chorioamnionitis. IUFD is known to be associated with a higher risk of shoulder dystocia of 1.1% as compared to 0.2% in the general population [1, 6]. One of the reasons is the lack of muscle tone of the dead fetus, so that descent of the fetal head and its shoulders were not synchronized. The shoulders remained above the pelvic inlet, and conventional manoeuvres failed to relieve dystocia [6, 7]. Lack of muscle tone also caused asynclitism leading to obstruction in our case.

Posterior axillary sling traction (PAST) was first reported by Cluver et al. in 2009, who used a soft plastic catheter as a sling in two cases of IUFD complicated with shoulder dystocia [5]. Subsequently, they reported 19 cases of PAST including 5 antenatal stillbirths and 14 livebirths, and all achieved successful birth. The 14 livebirths survived with one suffered from permanent anterior Erb’s palsy, which could be due to other manoeuvres applied before the sling method [7]. Advantages of PAST include easy manipulation, readily available material and simplicity of the technique. It is also easier to insert a sling than the whole hand in a limited space.

We modified the sling method by using two long ribbon gauzes tied together and a right-angle forceps for several advantages. While neonatal suction tubes or Foley catheters have to be folded to form a loop and then used as a sling, the knot of the ribbon gauze can be easily held between the fingers and brought to the fetal axilla [5, 7–10] (Fig. 1). More importantly, as ribbon gauzes are non-elastic, lesser force is required for traction when compared to using elastic rubber catheters. With less tension around the axilla, it is less likely to result in fetal injury, such as a recently reported case of circumferential shoulder laceration caused by a plastic sling [10]. We recommend to bring the gauze anteriorly and to use a right-angle forceps to grasp it posteriorly (Fig. 2a and b). The long and slim design of the right-angle forceps allows it to pass through a narrow space easily and to reach the fetal axilla even if it is located in the upper pelvis. Its curved, small but blunted tips also allow easy manipulation in the small and obscured area under the fetal axilla, as well as easy palpation by the operator’s fingers on the other side. We recommend birthing suites to maintain a stock of these two items in case the urgent need arises. While Cluver et al. used the sling primarily for traction, followed by shoulder rotation if traction had failed, we emphasize simultaneous traction and rotation, so that the shoulders are extracted through the wider oblique pelvic outlet dimension [5, 7] (Fig. 2d and Video 1).

As our modified PAST is simple to perform, we believe that this sling method can be safely applied on a live fetus with shoulder dystocia to shorten the head-to-body birth interval, which is a determining factor for hypoxic-ischemic brain injury [2]. Unlike rotational manoeuvres or posterior arm extraction, the operator does not need to insert hands deep into the pelvis to exert force on the fetal shoulders, or to flex the elbow to reach the forearm [5, 7]. The only prerequisite is to use two fingers to bring the knot to the fetal axilla. The blunt-end right-angle forceps is also non-traumatic. As the pulling force is exerted using the sling underneath the axilla, injury to the brachial plexus is minimal. The direction of the force aims at rotating the shoulder to the wider diagonal diameter of the maternal pelvis.

In conclusion, we concur with Cluver et al. that PAST is a safe and effective technique, especially in cases of shoulder dystocia with limited space for manipulation [7]. We modified the method by forming the sling with a ribbon gauze which has advantages over a rubber catheter, and with the assistance of a long right-angle forceps.

## Supplementary Information

**Additional file 1.**

## Data Availability

All data generated during this study are included in this case report.

## Abbreviations

IUFD: Intrauterine fetal death
PAST: Posterior axillary sling traction
